# Competition between plant and bacterial cells at the microscale regulates the dynamics of nitrogen acquisition in wheat (*Triticum aestivum*)

**DOI:** 10.1111/nph.12405

**Published:** 2013-07-12

**Authors:** David L Jones, Peta L Clode, Matt R Kilburn, Elizabeth A Stockdale, Daniel V Murphy

**Affiliations:** 1 Environment Centre Wales, Bangor UniversityGwynedd, LL57 2UW, UK; 2 Centre for Microscopy Characterisation and Analysis, University of Western AustraliaCrawley, WA, 6009, Australia; 3 School of Agriculture, Food and Rural Development, Newcastle UniversityNewcastle upon Tyne, NE1 7RU, UK; 4 Soil Biology and Molecular Ecology Group School of Earth and Environment, Institute of Agriculture, University of Western AustraliaCrawley, WA, 6009, Australia

**Keywords:** amino acids, dissolved organic nitrogen, NanoSIMS, nitrogen cycling, nutrient uptake, rhizobacteria, rhizosphere architecture

## Abstract

- The ability of plants to compete effectively for nitrogen (N) resources is critical to plant survival. However, controversy surrounds the importance of organic and inorganic sources of N in plant nutrition because of our poor ability to visualize and understand processes happening at the root–microbial–soil interface.
- Using high-resolution nano-scale secondary ion mass spectrometry stable isotope imaging (NanoSIMS-SII), we quantified the fate of ^15^N over both space and time within the rhizosphere. We pulse-labelled the soil surrounding wheat (*Triticum aestivum*) roots with either 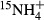 or ^15^N-glutamate and traced the movement of ^15^N over 24 h.
- Imaging revealed that glutamate was rapidly depleted from the rhizosphere and that most ^15^N was captured by rhizobacteria, leading to very high ^15^N microbial enrichment. After microbial capture, approximately half of the ^15^N-glutamate was rapidly mineralized, leading to the excretion of 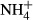, which became available for plant capture. Roots proved to be poor competitors for ^15^N-glutamate and took up N mainly as 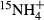. Spatial mapping of ^15^N revealed differential patterns of ^15^N uptake within bacteria and the rapid uptake and redistribution of ^15^N within roots.
- In conclusion, we demonstrate the rapid cycling and transformation of N at the soil–root interface and that wheat capture of organic N is low in comparison to inorganic N under the conditions tested.

Using high-resolution nano-scale secondary ion mass spectrometry stable isotope imaging (NanoSIMS-SII), we quantified the fate of ^15^N over both space and time within the rhizosphere. We pulse-labelled the soil surrounding wheat (*Triticum aestivum*) roots with either 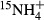 or ^15^N-glutamate and traced the movement of ^15^N over 24 h.

Imaging revealed that glutamate was rapidly depleted from the rhizosphere and that most ^15^N was captured by rhizobacteria, leading to very high ^15^N microbial enrichment. After microbial capture, approximately half of the ^15^N-glutamate was rapidly mineralized, leading to the excretion of 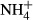, which became available for plant capture. Roots proved to be poor competitors for ^15^N-glutamate and took up N mainly as 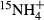. Spatial mapping of ^15^N revealed differential patterns of ^15^N uptake within bacteria and the rapid uptake and redistribution of ^15^N within roots.

## Introduction

Nitrogen (N) is the primary nutrient limiting plant productivity and frequently represents a major constraint to food production world-wide. Consequently, there is increasing interest in plant-based strategies that may promote N use efficiency in crop plants. Conventionally, it was thought that non-N_2_-fixing plants acquire their N from the soil as nitrate (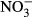 ) and ammonium (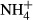; Loomis & Connor, 1992; Mosier *et al*., 2004). There is now strong evidence to suggest that, particularly in N-limiting environments, plants can directly acquire significant amounts of N in the form of organic N (e.g. amino acids and peptides) released during the breakdown of soil organic matter (Näsholm *et al*., 2009; Hill *et al*., 2011). However, the evidence to support the significance of organic N uptake in both natural and agricultural systems has drawn severe criticism (Sauheitl *et al*., 2009). This controversy is largely associated with limitations in experimental design (e.g. the inability to remove soil and microbial contamination from roots), the inappropriate interpretation of ^15^N and ^13^C isotopic data (e.g. lack of consideration of ^15^N pool dilution and N efflux from roots), poor consideration for differential carbon (C) and N fractionation in plants (e.g. metabolic and root–shoot partitioning) and the use of inappropriate N concentrations and timescales (Jones *et al*., 2005a,2005b; Rasmussen *et al*., 2010; Warren, 2012). While it is clear that plants can potentially take up and assimilate a wide range of organic N sources in sterile hydroponic culture (amino acids, peptides, proteins, urea, polyamines etc.; McKee, 1962; Jones & Darrah, 1994; Soper *et al*., 2011), it is the magnitude of the organic N flux versus 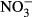 and 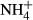 that is critical to determining its significance in natural systems. The simple demonstration that organic N is taken up intact from soil by the root also remains controversial (Sauheitl *et al*., 2009). Amino acids can be cleaved outside the root by extracellular enzymes or microorganisms releasing keto acids, 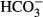 and 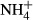, all of which can be taken up by the root but by different transport mechanisms. Some of the most convincing evidence for the direct uptake of organic N has come from experiments involving the use of dual ^13^C- and ^15^N-labelled amino acids, where isotopic compound-specific tracking has show the flow of exogenously applied ^13^C-^15^N-glycine through the plant (Persson & Näsholm, 2001; Thornton, 2001).

Our current understanding of N uptake in plants has been severely limited by the lack of suitable methods for the *in situ* imaging and quantification of isotopic N flow at the submillimetre scale. We have therefore had to rely on large bulk analysis of plants by conventional mass spectrometry, which prevents differentiation between adjacent tissues and between root cells and closely associated organisms (e.g. between epidermal and cortical cells, between roots and endophytic bacteria or between roots and their mycorrhizal symbionts). However, it is precisely at the root–soil interface and at this scale of resolution that experiments need to be conducted to resolve the controversy surrounding inorganic versus organic N nutrition. Secondary ion mass spectrometry (SIMS) enables imaging and isotopic discrimination for stable isotopes (e.g. ^14^N versus ^15^N), thereby providing the possibility to undertake spatially discrete studies of nutrient flow at the subcellular level. In a preliminary study, we showed the potential to spatially resolve ^15^N in wheat (*Triticum aestivum*) root cells through application of nano-scale SIMS (NanoSIMS; Clode *et al*., 2009). Here, we utilized a combination of traditional and NanoSIMS techniques to image and quantify competition for different forms of ^15^N *in situ* within the rhizosphere of wheat.

Our specific aims were to determine: the relative uptake rate of organic and inorganic N supplied to roots *in situ*; subsequent N partitioning within root tissues and within the whole plant; and the amount of competition for the different N forms from bacteria living on the rhizoplane and in the ecto- and endo-rhizosphere.

## Materials and Methods

### Microcosm preparation and ^15^N labelling

A coarse-textured agricultural soil routinely used for wheat production in rotation with legumes and dominated by quartz sand grains (92% sand) was obtained from a freely draining soil located in Meckering, Western Australia (31°40′N, 117°00′E). Soil samples were collected from the Ap horizon (0–15 cm; 10 ± 1 mg organic C kg^−1^ and 0.45 mg total N kg^−1^) using a stainless steel corer and stored in CO_2_-permeable polypropylene bags for transport back to the laboratory where they were sieved (< 5 mm) and stored field-moist at 3°C. Seeds of wheat (*Triticum aestivum* L.) were soaked for 24 h in water and then allowed to germinate on moistened filter paper at 20°C. After 3 d, each plant had one main root axis *c*. 1 cm in length and two lateral roots 0.5 cm in length; the seedlings were then placed into individual soil-filled microcosms (Owen & Jones, 2001). The plant–soil microcosms were constructed from polyethylene tubing (30 cm long; 0.6 cm internal diameter) and filled with soil to a bulk density of 1.25 g cm^−3^ and wetted to 70% of their water-holding capacity with distilled water. To half the microcosms, 3-d-old seedlings were added to the top and covered with a further 1 cm of soil. Planted and unplanted microcosms were then maintained at 20°C, with a light intensity of 260 μmol m^−2^ s^−1^ photosynthetically active radiation and a 12-h photoperiod. Soil within the microcosms was kept moist by the daily addition of deionized water. A large number of microcosms were set up and the ones with poor plant growth (< 10% total) were discarded before the microcosms were randomly pre-allocated to treatments and labelled. When the roots and associated root hairs had completely occupied the microcosm, making it essentially all rhizosphere soil (15 d after transplantation; shoots 12.4 ± 0.5 cm long; mean ± SE, *n *=* *12), 500 μl of 45 mg N l^−1^ (3 mM) 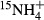 or ^15^N-labelled glutamate (0.99 ^15^N : ^14^N ratio; Isotec, Miamisburg, OH, USA) was injected at three locations (3, 5 and 7 cm below the soil surface) through pre-made holes, giving a total injection volume of 1500 μl. The volume of solution used was sufficient to occupy soil pores to *c*. 1 cm either side of the injection point. All microcosms were injected by the same person to avoid bias. The tubes were then randomized for microcosm harvesting and maintained at 20°C as described above. As controls, microcosms with and without plants were injected with 18 MΏ deionized water instead of ^15^N using the same injection procedures as described above. Glutamate was chosen as a model amino acid as it is central to amino acid metabolism, is present at high concentrations in plant tissues and soil organic matter (Friedel & Scheller, 2002; Gattolin *et al*., 2008), can be the dominant free amino acid in soil solution (Jones *et al*., 2005a,2005b) and is known to be taken up by plant roots (Jones & Darrah, 1994; Forsum *et al*., 2008).

### Microcosm harvesting

After 5, 30, 90, 360 or 1440 min of exposure to 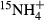 or ^15^N-glutamate, the microcosms were destructively harvested using three people to ensure rapid sample processing. The control, non-^15^N-labelled microcosms were harvested after 5 and 1440 min. Four independent microcosms were used for each harvest time for both the ^15^N-labelled substrates and the control treatment (i.e. *n *=* *4 replications). At each harvest time, the shoots were excised and dried at 80°C, weighed and stored at 20°C for isotopic analysis. Immediately following recovery of the shoots, 2-cm-long microcosm soil sections were cut from around each of the three injection points using a razor blade to allow rapid cutting with minimal disturbance. The three sections were randomly allocated for NanoSIMS analysis, soil solution recovery, or KCl extraction and root recovery. Immediately after excision, the 2-cm-long microcosm sections destined for NanoSIMS analysis (microcosm harvest times* *=* *5, 30 or 1440 min) and containing intact plant roots and soil were covered across the bottom with a cotton membrane, porous to water and solvents, which prevented soil movement during handling. These intact cores were then immersed in 2.5% glutaraldehyde at 4°C to enable extremely rapid fixation and then stored in 2.5% glutaraldehyde. The cross-linking nature of glutaraldehyde results in this chemical fixative being suitable for both the preservation and immobilization of ^15^N-labelled compounds in cellular material before NanoSIMS analysis, as demonstrated in Mays *et al*. (1984), Peteranderl & Lechene (2004), Herrmann *et al*. (2007), Clode *et al*. (2009) and Pernice *et al*. (2012). The time from excision to fixation was < 30 s and samples were stored in the glutaraldehyde for up to 24 h to ensure complete fixation of the soil core. An additional experiment performed on replicate 7-d-old wheat plants (*n *=* *4) grown in hydroponic culture (200 μM KCl and 1 mM CaCl_2_) and then supplied with ^15^N-labelled glutamate for 30 min or 24 h also showed no significant differences in ^15^N content between roots that had undergone fixation and dehydration (as above) and roots that had not (30 min, *P *=* *0.913; 24 h, *P *=* *0.09).

For soil solution recovery, the 2-cm-long microcosm section was weighed and then placed into a 1.5-ml Eppendorf tube in which a hole had been drilled in the bottom. This was then placed in the top of another 1.5-ml Eppendorf tube and the device was spun at 18 000 ***g*** for 5 min. The soil solution recovered in the lower Eppendorf tube was removed and respun at 18 000 ***g*** for 5 min, and the supernatant was stored at −20°C to await chemical analysis. The soil remaining was reweighed and dried overnight at 80°C and its dry weight determined. The soil solution recovered accounted for 83 ± 2% (mean ± SE) of the initial soil water present (264 ± 6 g H_2_O kg^−1^, mean ± SE).

The final 2-cm section was weighed and placed in 10 ml of 2 M KCl and shaken for 1 h on a rotary shaker at 60 rev min^−1^. After shaking, the excised roots were recovered and any loosely adhered soil was removed using forceps under a dissecting microscope and the samples were dried at 80°C, weighed and stored at 20°C for subsequent isotopic analysis. The 2 M KCl extract was then filtered through a GF-A glass fibre filter paper (vacuum-assisted), the residual soil was washed with 10 ml of 18 MΏ deionized water and the wash water was added to the 2 M KCl extract. The extracts were subsequently stored at −20°C to await analysis. The residual soil on the filter paper was recovered, using a small amount of 18 MΏ deionized water, into a Petri dish and the soil suspension was dried at 40°C (16 h) before weighing and storage at 20°C.

The amount of 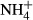 in soil solution and in the KCl extracts was determined using the hypochlorite-salicylic acid procedure of Mulvaney (1996) on a Cary Microplate Reader (Perkin-Elmer Corp., Beverly, MA, USA). Free amino acids were determined fluorometrially on a Cary Eclipse fluorimeter according to the *o-*phthialdehyde-β-mercaptoethanol procedure (Jones *et al*., 2002). Nitrate was determined colorimetrically on a San^+^ segmented flow autoanalyzer equipped with a Cd-Cu reduction column (Skalar Ltd, Breda, the Netherlands).

Quantification of 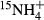 and 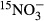 in the KCl extracts was performed using a two-stage microdiffusion over 7-d periods at 20°C. This was achieved by first diffusing 5 ml of the extracts with MgO, and subsequently with Devarda’s alloy (Brooks *et al*., 1989), and capturing separate ^15^N : ^14^N ratios in diffusion discs which were analysed by Tracer MS (Europa 20 : 20; Europa Scientific, Crewe, Cheshire, UK). Standards of ^15^N-enriched (NH_4_)_2_SO_4_ at 2 and 5 atom% excess were also diffused. Control (spiked) samples revealed no interference of ^15^N-glutamate in 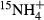 or 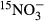 determination.

### Adsorption isotherms and amino acid turnover

To determine the soil-to-solid phase partitioning of 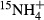 and ^15^N-glutamate after addition to the soil, concentration-dependent adsorption isotherms were performed. Briefly, solutions of ^14^C-glutamate (0.168 kBq ml^−1^) or 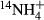 (0–5 mM; 10 ml) in a background of 0.01 M KCl were mixed with replicate (*n *=* *3) batches of soil (5 g) for 15 min (200 rev min^−1^), the soil suspensions were centrifuged (18 000 ***g*** for 5 min) and the amount of 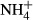 in the supernatant solution was determined as described in the previous section. The concentration of ^14^C-glutamate was determined using HiSafe 3® liquid scintillation fluid (Perkin-Elmer Inc., Waltham, MA, USA) and a Wallac 1408 liquid scintillation counter (Perkin-Elmer). To prevent microbial degradation of the added N sources, the soils were pre-sterilized at 80°C (for 30 min) before performing the assays (Kuzyakov & Jones, 2006). Our previous studies have shown that without this mild heat sterilization both N forms would be rapidly consumed by the soil microbial community, severely biasing the results (Rousk & Jones, 2010; Abaas *et al*., 2012). While heating caused no observable change in soil structure, we acknowledge that heating may cause the release of potentially competing amino acids from within microbial cells; however, we expect this to be minimal based on the large concentration of our added N in comparison to that present in the microbial community (Jörgensen, 1996). No significant effect of heating on the cation exchange properties of the soil was expected to occur (Forgeard & Frenot, 1996).

Glutamate mineralization in soil was determined as described in Hill *et al*. (2012). Briefly, ^14^C-U-glutamate (3 mM) was added to soil obtained from planted and unplanted microcosms (50 μl g^−1^) and ^14^CO_2_ captured over a 24-h period using 1 M NaOH traps. ^14^C in the traps was quantified by liquid scintillation counting using HiSafe 3® scintillation fluid and a Wallac 1408 scintillation counter. A double first-order kinetic model was fitted to the mineralization data and the first kinetic phase used to estimate the half-life of the ^14^C-glutamate added to the soil (see Boddy *et al*. (2008) and Hill *et al*. (2012) for further details of the modelling protocols). As the concentration of the introduced ^14^C-glutamate was much higher than the intrinsic ^12^C-glutamate concentration, the half-life estimates were not subject to major problems of isotopic pool dilution (Cobelli *et al*., 2001).

### Preparation and imaging of samples

Preparation and imaging of samples before NanoSIMS analyses were performed as described in detail by Herrmann *et al*. (2007) and Clode *et al*. (2009). Briefly, microcosm sections were fixed and stored in 2.5% glutaraldehyde, before being dehydrated with acetone and infiltrated with araldite. Resin-embedded soil plus plant root cores were cut into slices and re-embedded into 10-mm NanoSIMS mounts. These mounts were polished and gold-coated before being imaged at 15 kV using scanning electron microscopy (SEM; Zeiss 55 field emission) to identify regions of interest (ROIs) for NanoSIMS analysis. For correlative structural and NanoSIMS imaging of bacteria associated with root cells, fixed roots from the microcosms were extracted from the soil, dehydrated and resin-embedded. Thin (120-nm) sections were cut on a diamond knife and mounted on C-filmed copper grids, and ROIs were identified and imaged at 120 kV in a transmission electron microscope (TEM; JEOL 2100) fitted with a digital camera (Gatan, ORIUS1000; Gatan Inc., Pleasanton, CA, USA).

### NanoSIMS analyses

*In situ* isotopic mapping was performed using a NanoSIMS 50 (Cameca, Gennevilliers, France), with a 16-kV Cs^+^ primary ion beam. The analyses were performed in multi-collection mode, with the trolleys positioned to detect the negative secondary ions ^12^C^−^, ^16^O^−^, ^12^C^14^N^−^, ^12^C^15^N^−^, and ^28^Si^−^, simultaneously. The mass spectrometer was tuned to a high mass resolution of 9000 (CAMECA definition) to separate the ^12^C^15^N^−^ from the ^13^C^14^N^−^ peak on mass 27 using an entrance slit of 30 μm, an aperture slit of 200 μm, and a 10% reduction in the signal at the energy slit.

For standard resolution images, the primary current was set to *c*. 5 pA using a 300-μm primary aperture (D1), giving a spot size of *c*. 120 nm. Images were acquired by rastering the beam over an area 28 × 28 μm square, at a resolution of 256 × 256 pixels, giving a pixel size of 110 nm, slightly smaller than the beam diameter. For all samples, a systematic approach was taken to obtain comparable images from discrete distances extending from the root centre out into the rhizosphere – up to 500 μm. Poor preservation of root hairs and epidermal cells prevented their study in detail. High-resolution images of the sample prepared for TEM analysis were acquired with a *c*. 0.1-pA primary beam with a spot size of < 70 nm, using a smaller primary aperture (150 μm) and high voltage on the L1 lens. Initially, the same thin section samples were imaged in both TEM and NanoSIMS, but the samples were found to be extremely delicate under the high-energy ion beam, which would often ‘burn though’ after only one or two images had been acquired. Most of the isotope data for the bacteria were obtained from 1-μm-thick serial sections mounted on gold-coated aluminium stubs, with the adjacent thin sections prepared for TEM analysis, allowing the same bacteria and cortical cell walls to be imaged by both techniques. Image data consist of the total number of counts for a given secondary ion species recorded on each pixel, with count times kept constant at 40 ms pixel^−1^. All areas were implanted to the same ion dose by the primary beam before each acquisition to remove surface contamination and to enhance the generation of secondary ions.

Isotope data were extracted from the images using the openmims data analysis software (NIH/NIBIB National Resource for Imaging Mass Spectrometry, Cambridge, MA, USA) for the freeware package imagej (National Institute of Mental Health, Bethesda, MD, USA). Images were corrected for detector dead time (44 ns) on the individual pixels before any other data processing. Maps representing the ^15^N : ^14^N ratio were obtained by dividing the ^12^C^15^N counts by ^12^C^14^N counts on each pixel. Using the Hue-Saturation-Intensity function of the openmims data analysis software, it is possible to express the level of isotopic turnover in different components within a single image on a colour scale, thus highlighting the small, subtle differences that are not so apparent in a typical greyscale ratio image. Numerical ^15^N : ^14^N ratio data were extracted directly from the images by drawing ROI, discrete groups of pixels that define a particular feature, and extracting the total number of counts for the given ROIs. The ratio of the ROIs and the associated uncertainty (Poisson counting error) is then determined. The relative abundance of ^15^N : ^14^N from ROIs was then statistically compared with natural ^15^N abundance using Wilcoxon statistics. Counting errors for any given ROI were of the order of a few per cent. Quoted uncertainties are the standard error of the mean of the measured ROIs for any given bacterial/plant cell component, and were also of the order of a few per cent. This indicates that the reproducibility is tracking the counting statistics.

### ^15^N analysis of shoots, roots and soil

The entire shoots or roots for each replicate were weighed (5 decimal places; d.p.) into individual tin capsules for ^15^N determination. As the root samples were very small, a reference material (ANCA 56 Jarrah litter %N = 0.693; δ^15^N* *=* *−2.71; D.V. Murphy, University of Western Australia, WA, USA) was also weighed (5 d.p.) into the tin cups (*c*. 20 mg) to increase the N content and/or reduce the enrichment to within the range suitable for mass spectrometry. Tin cups receiving only the reference material were included to allow for back calculation of the reference material ^15^N : ^14^N ratio from the sample ^15^N : ^14^N ratio. Soil samples were ground using a mill adapted to the complete recovery of very small samples and subsequently weighed (*c*. 180 mg) into tin capsules for analysis. ^15^N in the prepared samples was determined using isotope ratio mass spectrometery (ANCA-NT system 20/20; Europa Scientific).

### Statistical analysis

Linear regression analysis of uptake rates and first-order kinetic analysis of the mineralization data were undertaken with sigmaplot v12 (SPSS Inc., Chicago, IL, USA). *t*-tests and ANOVA (with Tukey pair-wise comparison) were undertaken with minitab v16 (Minitab Inc., State College, PA, USA). *P *<* *0.05 was used as the upper limit for statistical significance.

## Results

### Nitrogen dynamics in the rhizosphere

**Figure 1:**
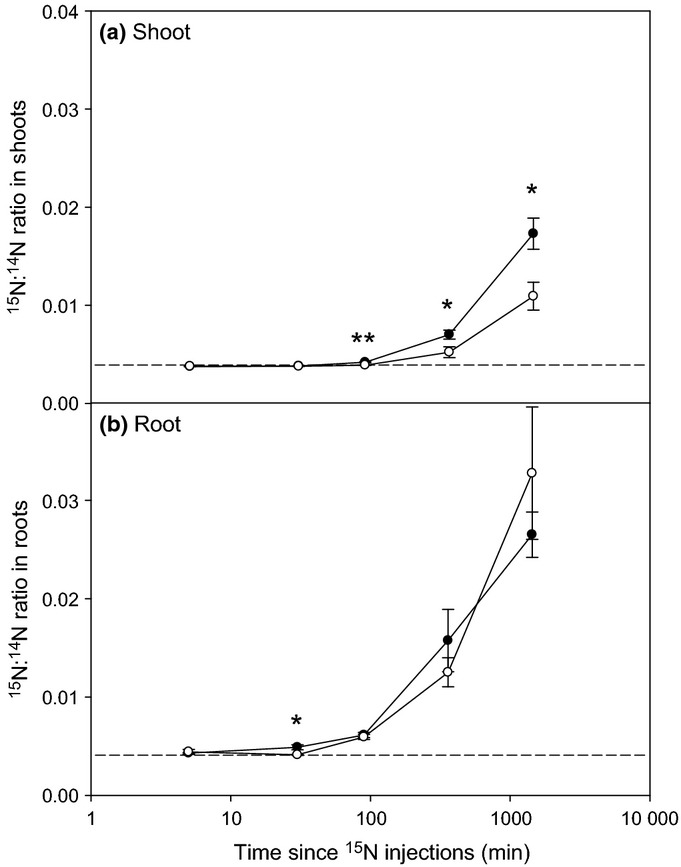
^15^N accumulation in the (a) shoots and (b) roots of wheat (*Triticum aestivum*) after the addition of ^15^NH_4_ (closed circles) or ^15^N-glutamate (open circles) to the rhizosphere. Dotted lines represent ^15^N natural abundance values in nonisotopically labelled plants. Values represent mean ± SE (*n *=* *4 replicate microcosms). Significant differences between the ^15^NH_4_ and ^15^N-glutamate treatments: *, *P *<* *0.05; **, *P *<* *0.01.

The time-dependent uptake of ^15^N within the wheat shoots and roots (including any microbial cells still attached) derived from the added 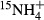 or ^15^N-glutamate is shown in Fig. 1. As expected, the rate of ^15^N uptake was fast and led to greater incorporation into root tissues in comparison to shoot tissues (*P *<* *0.05 for 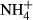 and glutamate). The first time-point at which significant ^15^N uptake could be detected in the roots relative to the background (microcosms not labelled with ^15^N) was 30 min for 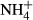, while for glutamate it was significantly later at 90 min. With respect to the shoots, significant ^15^N enrichment was seen in both N addition treatments after 90 min, relative to the unamended control (*P *<* *0.01). The rate of ^15^N uptake was linear in both treatments (*r*^2^ > 0.998), although the rate of ^15^N incorporation in the shoots was much greater from 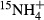 than from ^15^N-glutamate.

Overall, there were no significant differences in root biomass (mean ± SE across treatments was 22.7 ± 1.1 mg DW per plant), shoot biomass (mean ± SE across treatments was 21.5 ± 0.3 mg DW per plant) or root-to-shoot ratio over the 1440-min (24-h) experimental period or between the two N treatments and the water amended control microcosms (*P *>* *0.05). Similarly, there were no differences in root and shoot N contents between treatments or harvest times (root N, 1.29 ± 0.03% DW; shoot N, 5.6 ± 0.1% DW; mean ± SE, *P *>* *0.05).

### Nitrogen isotopic behaviour in the soil

**Figure 2:**
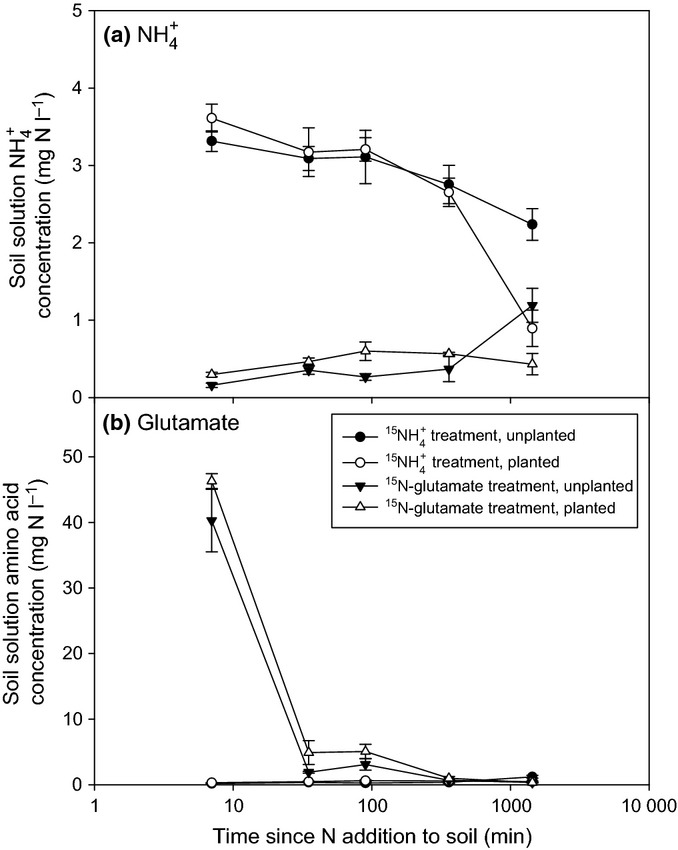
Concentration of nitrogen (N) in soil solution after the pulse-labelled addition of (a) 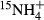 or (b) ^15^N-glutamate to soil in the presence and absence of wheat (*Triticum aestivum*) plants. The initial concentration was 45 mg N l^−1^. Because of the potential inter-conversion of N forms in soil, 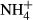 was also measured in the glutamate treatment and vice versa. Values represent mean ± SE (*n *=* *4 replicate microcosms). The legend is the same for both panels (a) and (b).

Adsorption isotherms with sterile soil revealed that added 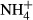 became preferentially bound to the soil’s solid phase (*c*. 85% of the total; Supporting Information Fig. S1). In relation to the initial concentration of 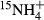 added to the microcosms (45 mg N l^−1^), and consistent with the adsorption isotherm data, there was a very rapid depletion of 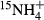 within the first 5 min to 3.5 mg N l^−1^ (Fig. 2a). The 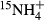 soil solution concentration then continued to decline, albeit at a much slower rate, over the subsequent 1440-min experimental period (Fig. 2a). By 1440 min, significantly greater amounts of 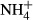 had been removed from soil solution in the planted microcosms in comparison to the unplanted microcosms (*t*-test *P *<* *0.05 at 24 h; Fig. 2a). Extraction of the soil over time with KCl (to quantify the solution and exchangeable phase, that is, the plant-available ^15^N pool) revealed that most 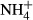 was not taken up by the plant in the first 360 min but remained bound to the soil’s solid phase (Fig. S2). By 1440 min, however, significant depletion of 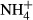 from the soil exchange phase had occurred in the planted microcosms.

In the amino acid treatment, ^15^N-glutamate was also rapidly depleted (within 30 min) from soil solution but at a similar rate in the planted and unplanted microcosms (Fig. 2b). At the pH of the soil (pH 4.8 ± 0.1, mean ± SE), the net charge on glutamate was predicted to be −0.66. Adsorption isotherms revealed no detectable sorption of glutamate to the soil’s solid phase (data not presented). Some mineralization of the glutamate-N also occurred, as evidenced by a net accumulation of 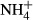 in the ^15^N-glutamate treatment, particularly in the absence of plants (Fig. 2a). The ^14^C-labelled glutamate was also shown to rapidly mineralize to ^14^CO_2_ after addition to both the planted and unplanted microcosms (Fig. S3). A double first-order kinetic model fitted well to the experimental mineralization data (*r*^2^* *=* *0.990 ± 0.002, mean ± SE) from which the half-life of the amino acid in soil solution was estimated to be 342 ± 36 min in planted and 708 ± 192 min in unplanted treatments (mean ± SE).

**Figure 3:**
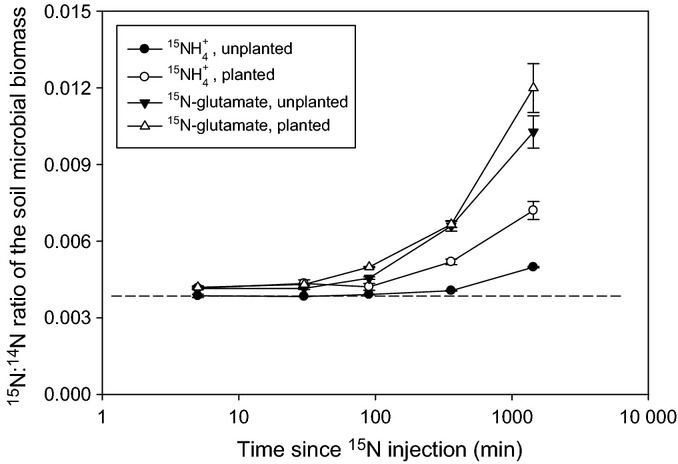
^15^N enrichment in the soil microbial biomass after the pulse-labelled addition of 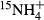 or ^15^N-glutamate to soil in the presence and absence of wheat (*Triticum aestivum*) plants. The dotted line represents ^15^N natural abundance in the unlabelled microcosms. Values represent mean ± SE (*n *=* *4 replicate microcosms).

After extraction of the ^15^N-amended soils with KCl and removal of roots, the ^15^N content of the residual soil was determined. We ascribe this pool to ^15^N immobilized in the soil microbial biomass (Jörgensen & Brookes, 1990; Murphy *et al*., 2003), the size of which was the same in planted and unplanted microcosms (220 ± 18 mg microbial biomass-C kg^−1^ soil; mean ± SE, *P *>* *0.05). The results presented in Fig. 3 indicate that both ^15^N forms became progressively immobilized by the microbial community over the 1440-min experimental period, although the rate of immobilization was much higher for ^15^N-glutamate than 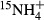 (*P *<* *0.001, unplanted; *P *<* *0.01, planted). The presence of plants had no influence on the rate of ^15^N-glutamate immobilization, whereas significantly greater immobilization occurred in the planted 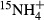 treatment (*P *<* *0.001).

The background concentrations of free amino acids, 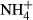 and 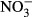 in both the planted and unplanted soils were very low (free amino acids 0.17 ± 0.01 mg N l^−1^; 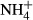 0.07 ± 0.01 mg N l^−1^; 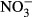 < 0.05 mg N l^−1^, mean ± SE), particularly in relation to the microcosms injected with ^15^N. The concentration of 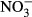 in soil solution remained low in both ^15^N treatments with no apparent conversion of ^15^N-glutamate or 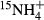 to 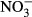 in any treatment at any harvest time (data not presented). Assuming perfect mixing of the added isotope with the intrinsic soil solution, we estimate that the initial isotopic pool dilution (i.e. by the ^14^N into the ^15^N pool) was very low, being 1.0% for 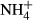 and 0.35% for glutamate. As a consequence of uncertainty over the native rates of ^14^N turnover and N gradients in the rhizosphere, it was not possible to estimate subsequent rates of isotopic pool dilution during the course of the experiment.

### NanoSIMS imaging and quantification

**Figure 4:**
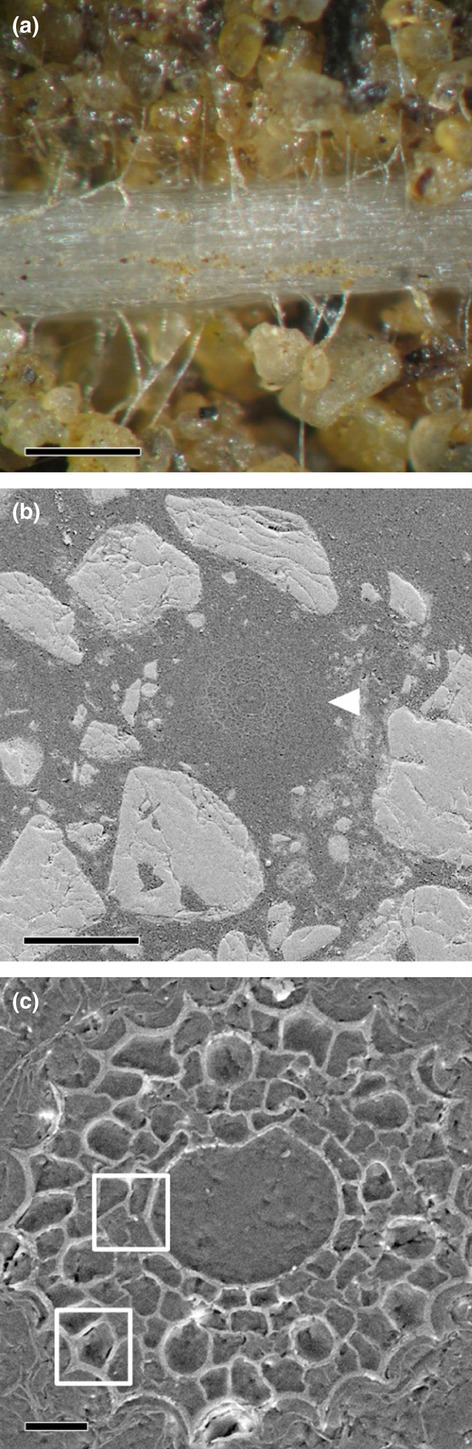
Imaging of plant roots *in situ*. (a) Optical micrograph of a wheat (*Triticum aestivum*) root growing within the microcosm. (b) Scanning electron micrograph (SEM) of a polished transverse section showing a plant root (arrow) within the embedded soil core. (c) SEM of a plant root within the embedded soil core at higher magnification, revealing the cellular root structure of inner cortical cells. Root hairs and epidermal cells cannot easily be seen in this image. Boxes indicate cellular regions typically analysed by nano-scale secondary ion mass spectrometry (NanoSIMS) (as seen in Fig. 6 and Supporting Information Fig. S4). Bars: (a) 400 μm; (b) 200 μm; (c) 20 μm.

**Figure 5:**
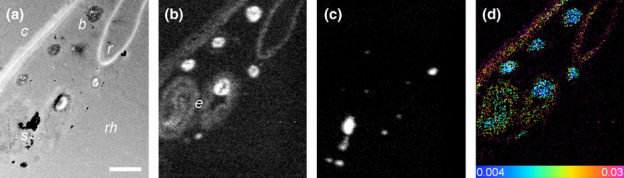
Correlative transmission electron micrograph (TEM) and nano-scale secondary ion mass spectrometry (NanoSIMS) imaging after 5 min of exposure of wheat (*Triticum aestivum*) to ^15^N-labelled 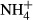. (a) TEM of the rhizosphere (*rh*), showing bacteria (*b*) adjacent to a root cell (*c*), root hair (*r*) and soil particles (*s*). (b) ^12^C^14^N^−^ NanoSIMS image of the same region, highlighting the bacteria and organic materials including extracellular mucilage (*e*) associated with soil particles and bacteria. (c) ^16^O^−^ NanoSIMS image of the same region highlighting soil particles containing oxides. (d) Hue-Saturation-Intensity (HSI) image generated from the NanoSIMS ^15^N : ^14^N ratio of the same region, showing ^15^N : ^14^N ratio intensities from natural abundance (blue, 0.0037) to enriched (pink, 0.30). Bar, 1 μm for all images.

**Figure 6:**
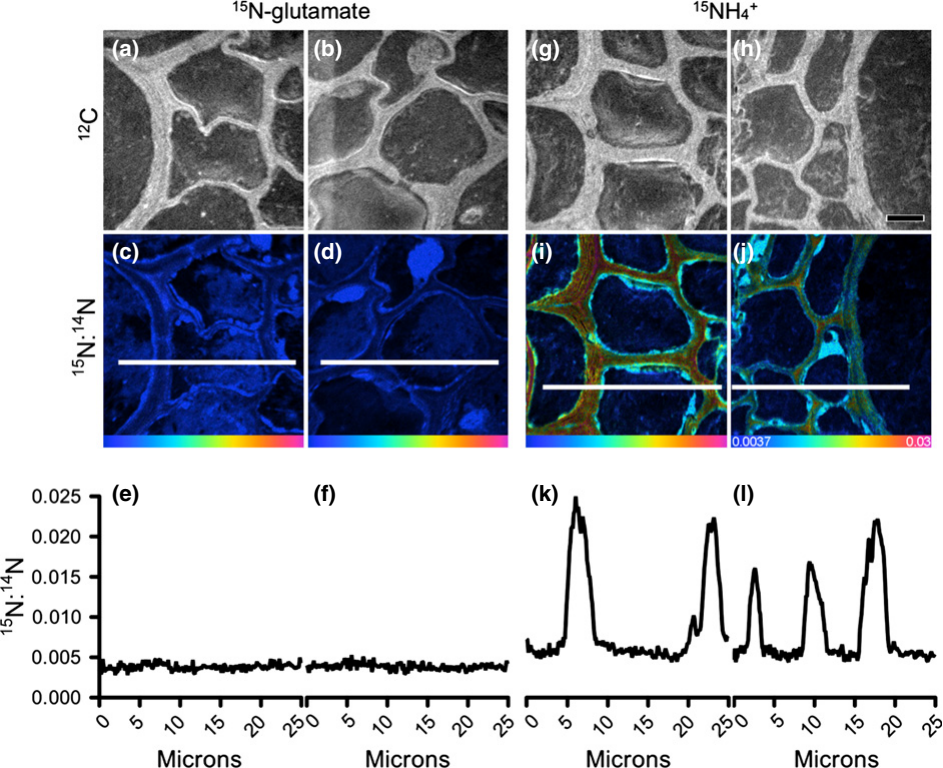
Nano-scale secondary ion mass spectrometry (NanoSIMS) imaging and analysis of wheat (*Triticum aestivum*) roots *in situ* within the embedded soil core after 5 min of exposure to ^15^N-labelled glutamate or 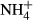. Examples of the location of cellular regions analysed within a root are depicted in Fig. 4(c). Root and cell structure is visualized in ^12^C images (a, b; g, h). Enrichment levels of ^15^N within these regions are shown as ^15^N : ^14^N Hue-Saturation-Intensity (HSI) images (c, d; i, j). The HSI colour scale (j; 0.0037–0.03; ^15^N : ^14^N natural abundance = 0.0037) applies to all HSI images. Line scans show numerical levels of ^15^N enrichment across the subcellular regions (e, f; k, l), with data acquired from the lines indicated on each of the respective ^15^N : ^14^N HSI images. High levels of ^15^N enrichment can be seen in the 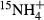 treatment within distinct cellular regions, whereas the ^15^N-glutamate treatment shows cells with very low levels of ^15^N enrichment just above natural abundance. Bar, 5 μm for all images.

Representative examples of roots within the experimental microcosms are presented in Fig. 4. The light micrograph shows the dense proliferation of root hairs, while the SEM images show cross-sections of the resin-embedded plant roots with the soil matrix. SEM or TEM images were used to aid identification of the specific location of cell components within the plant roots; these ROIs were subsequently analysed by NanoSIMS (Clode *et al*., 2009). The soil organic matter and mineral particles surrounding the root could also be easily distinguished using the ^12^C^14^N^−^ and ^28^Si^−^ (or ^16^O^−^) signals, respectively (Fig. 5). Areas of low signal strength correspond to resin-filled void spaces in the root (e.g. cell vacuoles) and soil (e.g. pore spaces); analyses of resin-only areas revealed that ^15^N abundance was at natural levels and had not been enriched. The cellular structure of the root, and particularly the stele with its thickened cell walls, could be readily distinguished in NanoSIMS images from transverse sections through a plant root embedded within the soil matrix (Figs 6, S4).

**Figure 7:**
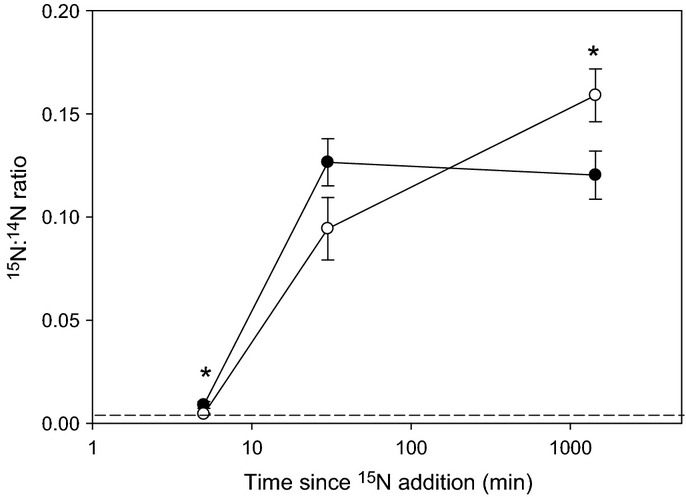
Nano-scale secondary ion mass spectrometry (NanoSIMS) derived ^15^N : ^14^N isotopic ratios of metabolically active (i.e. ^15^N-enriched) individual bacteria located in the rhizosphere of wheat (*Triticum aestivum*) plants after injection of 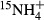 (closed circles) or ^15^N-glutamate (open circles) into the soil. The dotted line represents ^15^N natural abundance. Values represent mean ± SE across all four microcosms (*n *=* *11–50 regions of interest containing bacterial cells per time period for 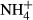 and *n *=* *29–36 for ^15^N-glutamate treatments). The dotted line represents ^15^N natural abundance in the non-^15^N-labelled treatment. Significant differences between the ^15^NH_4_ and ^15^N-glutamate treatments: *, *P *<* *0.05.

The isotopic enrichment of rhizosphere bacteria and root tissues normalized to unlabelled samples was determined for each treatment at the 5, 30 and 1440 min harvest times. Measurements of ^15^N : ^14^N ratios from corresponding regions of organic matter in unlabelled rhizosphere samples were equivalent to natural abundance, with a mean ^15^N : ^14^N ratio value of 0.003675 and standard error of 0.000015 (*n *=* *15), thus giving a precision of 1.6% (standard deviation = 0.000059). Overall, the temporal pattern of ^15^N uptake within the bacterial cells was similar between the two ^15^N treatments (Fig. 7). Despite this similar trend, initially (0–5 min) the amount of ^15^N uptake into the bacterial cells was significantly greater (*P *<* *0.05) in the 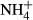 treatment (^15^N : ^14^N ratio data: mean and standard error = 0.009030 ± 0.000196; range = 0.003896–0.102855; *n* = 64 bacterial cells) compared with the glutamate treatment (^15^N : ^14^N ratio data: mean and standard error = 0.004657 ± 0.000269; range = 0.003807–0.01273; *n *=* *36 bacterial cells), with both enriched relative to natural abundance (Fig. 7). It should be noted that a few bacterial cells (as identified by TEM) that did not take up ^15^N were also present in the rhizosphere (Fig. 5); the mean ^15^N : ^14^N ratio values for data presented in Fig. 7 only include data from the active (i.e. ^15^N enriched) populations. No difference was apparent between the two N treatments after 30 min; however, by 1440 min, a very high proportion of ^15^N had accumulated in the bacterial cells relative to natural abundance, and this proportion was significantly higher in the glutamate treatment than in the 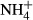 treatment.

**Figure 8:**
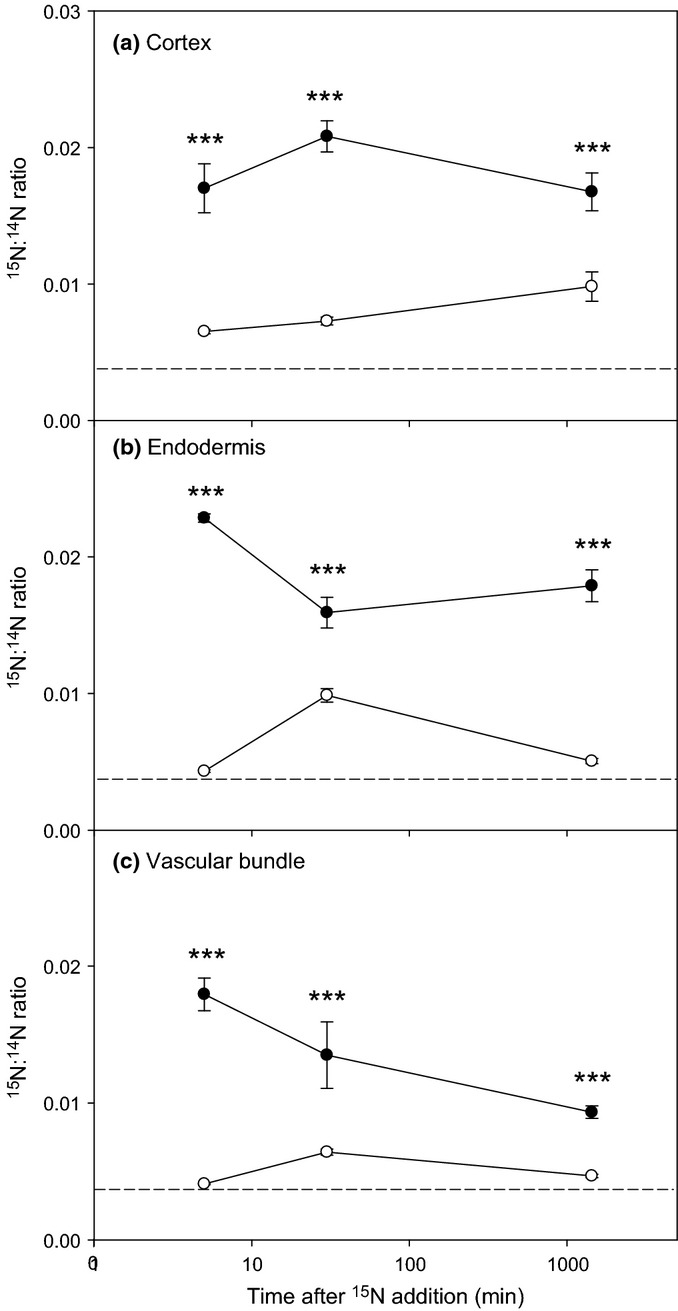
Nano-scale secondary ion mass spectrometry (NanoSIMS) derived ^15^N : ^14^N isotopic ratios for different wheat (*Triticum aestivum*) root tissues (intercellular cortex, endodermis and vascular bundle) after injection of 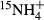 (closed circle) or ^15^N-glutamate (open circle) into the rhizosphere. Dotted lines represent ^15^N natural abundance. Values represent mean ± SE (*n *=* *13 and *n *=* *10 for roots in the 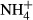 and glutamate treatments, respectively). The dotted line represents ^15^N natural abundance. Significant differences between the ^15^NH_4_ and ^15^N-glutamate treatments: ***, *P *<* *0.001.

Enrichment of ^15^N within subcellular regions of plant root cells was easily distinguishable by NanoSIMS (Fig. 6) and could be traced over time (Fig. S4). After 5 min there were significant differences in plant tissue ^15^N enrichment between treatments; ^15^N enrichment in the 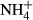 treatment was present across the entire root structure (Fig. 6), having reached the endodermis and vascular bundles (Fig. 8), whereas ^15^N enrichment in the glutamate treatment was only present in the outer cortical cells (Fig. 8). The levels of ^15^N enrichment in the plasma membrane of the plant tissues (Fig. 8) were generally 10-fold lower than observed in the rhizosphere bacteria (Fig. 7). In addition, the overall temporal pattern of ^15^N uptake was slightly different between the different tissue types (*P *<* *0.05 for both N types) and unlike that observed for the microbial cells. The level of ^15^N enrichment was significantly lower in the cortex after 1440 min relative to that present at 30 min in the 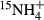 treatment (*P *<* *0.01). ^15^N enrichment in root tissues treated with ^15^N-glutamate was significantly lower than that of the 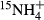 roots at all time-points (*P *<* *0.001; Fig. 8). Although cell nuclei (e.g. see Fig. S4c) could not be seen in all replicates and at all time-points, when sufficient nuclei could be enumerated they were also similarly ^15^N enriched in both the 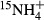 and ^15^N-glutamate treatments (^15^N : ^14^N ratio, 0.016 ± 0.001 at 1440 min, mean ± SE).

## Discussion

### Dynamics of ^15^N from a plant perspective

It has been demonstrated under field conditions that wheat has the potential to take up amino acids intact from soil, although its quantitative significance in terms of plant N nutrition remains highly uncertain (Näsholm *et al*., 2001, 2009). To assess this uncertainty, we took a microcosm approach to critically evaluate the temporal and spatial partitioning of ^15^N in different plant, microbial and soil pools. Our results showed an almost linear uptake of ^15^N by the wheat roots over the 1440-min labelling period and that, overall, plants competed better for 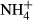 as a source of N in comparison to glutamate. Further, proportionally more N derived from 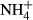 was allocated to the shoots in comparison to glutamate-derived N. This is in agreement with findings for wheat seedlings grown in sterile hydroponic culture, where a linear rate of N uptake was observed over a 360-min period and 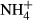 was also taken up preferentially in comparison to alanine, trialanine or 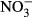 (Hill *et al*., 2011). We note that the plant–microbial competition scenario presented here is not reflective of severely N-limited ecosystems where N supply is regulated more by inputs of N from N_2_ fixation, atmospheric deposition and slow rates of organic matter turnover (e.g. Inselsbacher & Näsholm, 2012).

The type and amount of N taken up by plants are dependent on several factors, including the concentration of N in soil solution, its diffusion rate through soil, the activity and affinity of the root’s membrane transporters, the N status of the plant and the competition from microbes inhabiting the rhizosphere (Glass *et al*., 2002; Forsum *et al*., 2008; Jones *et al*., 2009). The soil used here was naturally low in available N and the amount of N added to the soil was chosen to reflect a pulse addition of N that would occur after the addition of an inorganic- or organic-based fertilizer or as a result of the lysis of plant cells in response to physical (e.g. abrasion or osmotic stress), chemical (e.g. metal-induced bursting of epidermal cells) or biological (e.g. toxin) stress (typically 2–30 mM 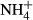 or free amino acids in root cell sap; Jones & Darrah, 1994; Miller *et al*., 2001). Measurements of soil N availability indicated a rapid depletion of the added glutamate and especially 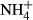 from the soil solution pool. However, the reasons for this disappearance were solute-specific, being mainly attributable to abiotic ion attraction to soil particles for 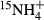 (i.e. cation exchange) and to biological immobilization for ^15^N-glutamate. This supports the findings of previous studies that have shown amino acids are rapidly mineralized by the soil microbial community, whose growth is more limited by C than N availability (Dempster *et al*., 2012). These findings also suggest that the timing of plant ^15^N uptake was significantly different between the two N treatments. Specifically, as 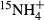 was not irreversibly bound to the soil’s exchange phase, it was capable of being readily released back into the available pool as the soil solution 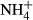 concentration fell as a result of progressive biotical removal. This indicates that a large reservoir of potentially available 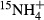 existed for the entire duration of our experiment. By contrast, after microbial uptake of almost all of the ^15^N-glutamate within 30 min, the plant-available ^15^N amino acid pool was effectively depleted (although roots may have taken up some unlabelled amino acids produced *de novo* from soil organic matter turnover). Thus, the period for root 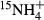 uptake was actually 50-fold longer than that for ^15^N-glutamate. Our results further indicate that the microbial community fed ^15^N-glutmate also effluxed a significant amount of N back into the soil as 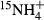 and that this process occurred within 5 min of glutamate addition in the planted microcosms. This 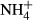 efflux process is induced by the internal catabolism of the added amino acids (in which 40% of the amino acid-C is typically respired) which causes a C : N ratio imbalance between the microbial biomass and the added glutamate, leading to the dumping of excess N (Jones *et al*., 2005a,2005b; Manzoni *et al*., 2008). Thus, our view is that plant ^15^N uptake in the glutamate treatment was actually a result of a small amount of intact amino acid uptake in the first 60 min followed by the uptake of microbially effluxed 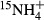 over the subsequent 1380-min (23-h) period. This is supported by Roberts *et al*. (2009) and the lack of reappearance of ^15^N-glutamate in soil solution after microbial uptake (Fig. 2). This may also explain the apparent lag phase in ^15^N uptake in the glutamate treatment in comparison to 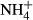 (as revealed by both NanoSIMS and conventional mass spectrometry in addition to the observed slower translocation of ^15^N to the shoots). This poor root capture of glutamate supports previous work showing that plants could only capture a very small amount of amino acid C supplied to nonsterile wheat roots (Owen & Jones, 2001).

Although 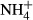 diffuses 50% faster in free solution than glutamate, we calculated that, because of its strong attraction to the solid phase, its effective linear rate of diffusion in soil over the 1440-min experimental period was fivefold slower than for glutamate (0.13 cm d^−1^ for 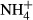 and 0.62 cm d^−1^ for glutamate). In our experimental set-up, the inter-root distance was *c*. 1–2 mm, implying that nearly all the 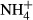 was available to the roots during the 1440-min experimental period. In other situations, if the inter-root distance were larger (*c*. 5 mm), then much of the 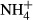 would have been incapable of diffusing towards the root within the experimental period. We hypothesize that this alternative scenario would additionally favour root capture of glutamate over 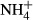.

At high exogenous concentrations (> 1 mM) in axenic soil-less culture, glutamate has been shown to be inhibitory to root growth, particularly when applied to the root apex (Sivaguru *et al*., 2003; Forde & Walch-Liu, 2009). In this soil-based study, however, the localized addition of glutamate revealed no negative effect of glutamate on either root or shoot growth. We ascribe this lack of negative response to the very rapid lowering of the glutamate concentration in solution by the soil microbial community (and to some extent root consumption), which lowered the glutamate concentration to below the critical response threshold. In addition, inhibitory effects of exogenously applied glutamate on root growth were only observed in hydroponic culture at concentrations > 5 mM (Fig. S5).

### Spatial distribution of ^15^N in plant tissues

Our results showed that the added ^15^N was quickly taken up and rapidly distributed throughout the root tissues (i.e. within 5 min of 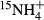 addition). Although we did not quantify ^15^N from root hair or epidermal cells (because of either poor preservation or lack of alignment in the field of view), our results suggest no preferential accumulation of ^15^N in any root cell type (there was never any accumulation of ^15^N within xylem tissue; e.g. Fig. S4). This supports the view that ^15^N is rapidly translocated away from the point of uptake to the active growing regions (e.g. root tips and shoots). In terms of root uptake, NanoSIMS did reveal the presence of ^15^N super-enriched bacteria in both the ecto-rhizosphere (Fig. 5) and endo-rhizosphere (Clode *et al*., 2009). It is likely that the typical root-washing procedure will not remove these bacteria and that these may have represented a significant contamination of the root ^15^N signal in many previous soil-based studies. As we injected ^15^N into root regions with fully expanded root hairs, this may favour the microbial capture of uptake of amino acids because of the higher microbial population in comparison to the low density of microorganisms surrounding root tips (Marschner *et al*., 2011). Further, the distribution and expression of root N transporters may be different between the actively and nonactively growing root regions (Yuan *et al*., 2007).

Another benefit of the NanoSIMS approach is that ^15^N in the apoplast can be readily distinguished from ^15^N present in the symplast (i.e. true uptake). At high external concentrations (> 1 mM), apoplastic diffusion of 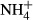 can readily take place until it reaches the endodermis, at which point the 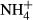 is taken up before translocation (Frensch *et al*., 1996; Yuan *et al*., 2007; Schreiber, 2010). Our NanoSIMS images indicated that some apoplastic ^15^N was present in the cortex at 5 min but that this extracellular pool was readily depleted to background levels by 30 min. Based on the equilibrium soil solution 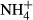 concentration (< 225 μM), we speculate that most 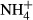 was taken up using high-affinity transporters in the root hairs and epidermis and that 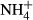 transport across the root to the stele occurred symplastically.

### Dynamics of ^15^N from a microbial perspective

NanoSIMS data revealed that initial bacterial uptake of 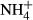 was faster than for glutamate but that the internal capacity for 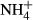 quickly became saturated (< 30 min), after which little 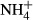 uptake occurred. By contrast, ^15^N-glutamate incorporation into the active bacterial community continued to rise throughout the experiment, suggesting that the added C was fuelling the formation of new cell biomass that necessitated the uptake of more N. These highly ^15^N-enriched bacteria were most often seen close to the root surface or in the endorhizosphere. We hypothesized that these rhizosphere bacteria would already be highly active and primed for glutamate uptake in response to the continual release of acidic amino acids from the roots into the soil via passive root exudation (Ayers & Thornton, 1968). Therefore, the lack of appreciable uptake of ^15^N derived from glutamate within the bacterial cells within 5 min was surprising. It is possible that wheat rhizodeposition contains low amounts of glutamate, as reported by Phillips *et al*. (2004), or that the bacterial transporters and internal metabolic capacity were initially saturated by the added glutamate pulse (Jones & Hodge, 1999). The latter is supported by the intrinsically low solution concentrations of total amino acids in this soil (12 ± 1 μM, mean ± SE), suggesting that the microbial community will have been operating high-affinity transport systems (Anraku, 1980). It should also be noted that we only investigated ^15^N uptake in rhizobacteria and not in the wider microbial community (i.e. fungi and actinomycetes). Although no distinct arbuscular mycorrhizal structures were observed in our roots when viewed by TEM, NanoSIMS or conventional light microscopy of trypan blue-stained roots, we cannot discount the potential for mycorrhizal transfer of ^15^N from the soil into the inner root tissues. The likelihood that this represents a major uptake N pathway in comparison to direct root uptake, however, is very low (Hawkins *et al*., 2000).

The C and N status of rhizosphere microorganisms remains an area of hot debate. Depending on the prevailing soil conditions, microbial growth could be C, N or phosphorus (P) limited (Denison *et al*., 2003; Marschner *et al*., 2011). Our 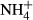 results obtained with NanoSIMS suggest that they were N limited in the short term and then became C limited. Previous work in sterile hydroponic culture has suggested that passive exudation is dominated by simple low-molecular-weight C compounds that are released in response to the large cytoplasmic–soil solution diffusion gradient (e.g. glucose and sucrose) or by compounds that are released to alleviate stress (e.g. organic acids). In both these cases we would expect this to lead to microbial N limitation in the rhizosphere, which would be exacerbated by root removal of available soil N. The rapid immobilization of 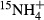 observed here would support this view.

### Conclusions

The rhizosphere represents a site of intense resource competition between plant roots and soil microorganisms. Here, we demonstrate that wheat roots represent poor competitors for glutamate in the soil in comparison to 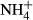 when both N forms are applied at the same rate. We ascribe this poor ability of the root to capture glutamate to the rapid immobilization and super-accumulation of amino acid-N in rhizobacteria, which prevents root uptake. This favouring of inorganic N by wheat roots may also have been exacerbated by the targeted breeding of wheat to favour the efficient acquisition of inorganic fertilizers (Reeve *et al*., 2009; Sadras & Lawson, 2013). Although the microbial community does immobilize 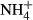, this was low in comparison to glutamate. After amino acid assimilation, our results suggest that the rhizosphere bacteria rapidly excrete excess 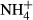 back into the soil, at which point it becomes available again for plant uptake. This study clearly demonstrates that N is rapidly transformed and cycled within the rhizosphere and highlights the extremely spatially localized nature of N dynamics at the plant–soil interface.

## Supporting Information

**Fig S1** Amount of  held on the soil’s solid phase as a function of solution  concentration. **Fig S2** Total amount of  present in soil solution and held on the soil’s cation exchange phase in *Triticum aestivum* planted and unplanted microcosms. **Fig S3** Time-dependent mineralization of ^14^C-labelled glutamate in soil in the presence and absence of wheat (*Triticum aestivum*) roots. **Fig S4** NanoSIMS imaging and analysis of wheat (*Triticum aestivum*) roots *in situ* within the embedded soil core after exposure to ^15^N-labelled glutamate uptake. **Fig S5** Influence of external solution glutamate concentration on the growth rate of wheat (*Triticum aestivum*) roots exposed to various concentrations of glutamate.
