# Supplementary material for: Competition between plant and bacterial cells at the microscale regulates the dynamics of nitrogen acquisition in wheat (Triticum aestivum)
Source: New Phytol. 2013 Jul 12;200(3):796–807. doi: 10.1111/nph.12405 (PMC3916831; doi:10.1111/nph.12405)
Supplement: Supplementary file 1 — Fig S1 Amount of held on the soil’s solid phase as a function of solution concentration. Fig S2 Total amount of present in soil solution and held on the soil’s cation exchange phase in Triticum aestivum planted and unplanted microcosms. Fig S3 Time-dependent mineralization of 14C-labelled glutamate in soil in the presence and absence of wheat (Triticum aestivum) roots. Fig S4 NanoSIMS imaging and analysis of wheat (Triticum aestivum) roots in situ within the embedded soil core after exposure to 15N-labelled glutamate uptake. Fig S5 Influence of external solution glutamate concentration on the growth rate of wheat (Triticum aestivum) roots exposed to various concentrations of glutamate. [file nph0200-0796-sd1.pdf]

Supporting Information Figs S1–S5

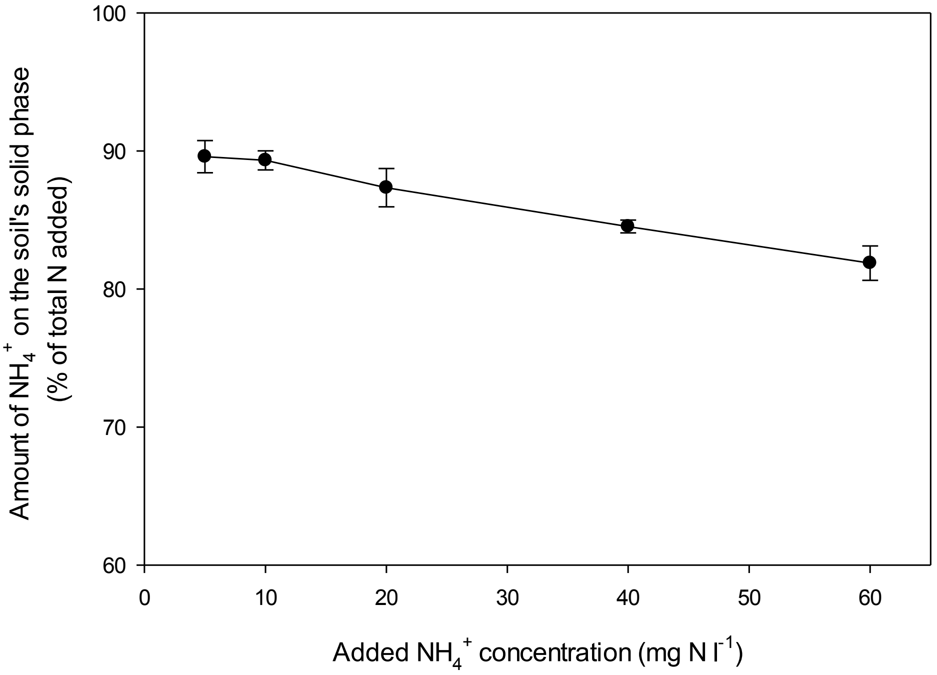

Fig. S1 Amount of NH_4^+ held on the soil's solid phase as a function of solution NH_4^+ concentration. Values represent means ± SEM (n = 3).

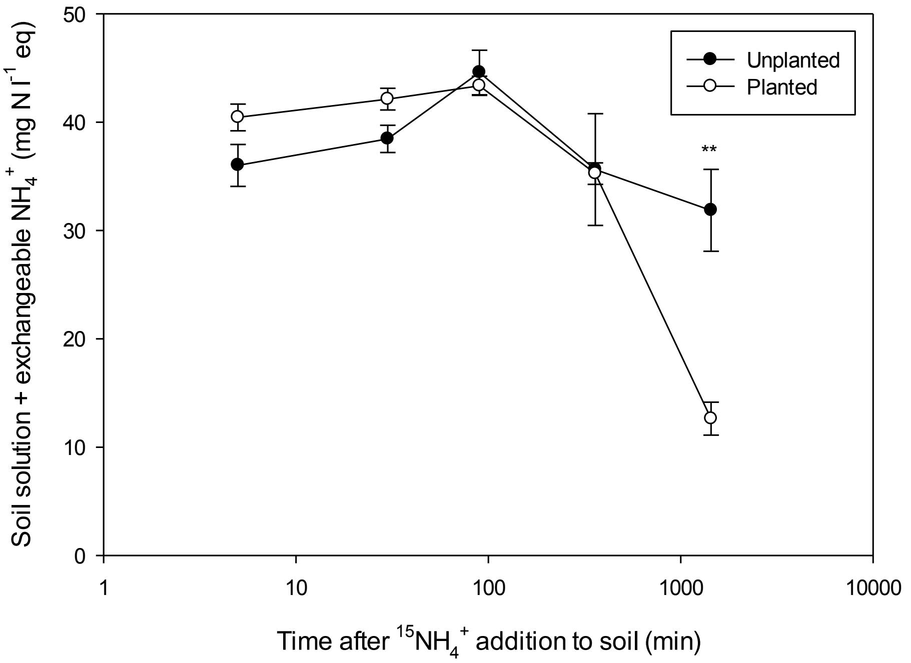

Fig. S2 Total amount of $^{15}NH_4^+$ present in soil solution and held on the soil's cation exchange phase in planted and unplanted microcosms to which $^{15}NH_4$ was injected. Values represent means ± SEM (n = 4). ** indicate significant differences between the planted and unplanted treatments at the $P < 0.01$ level.

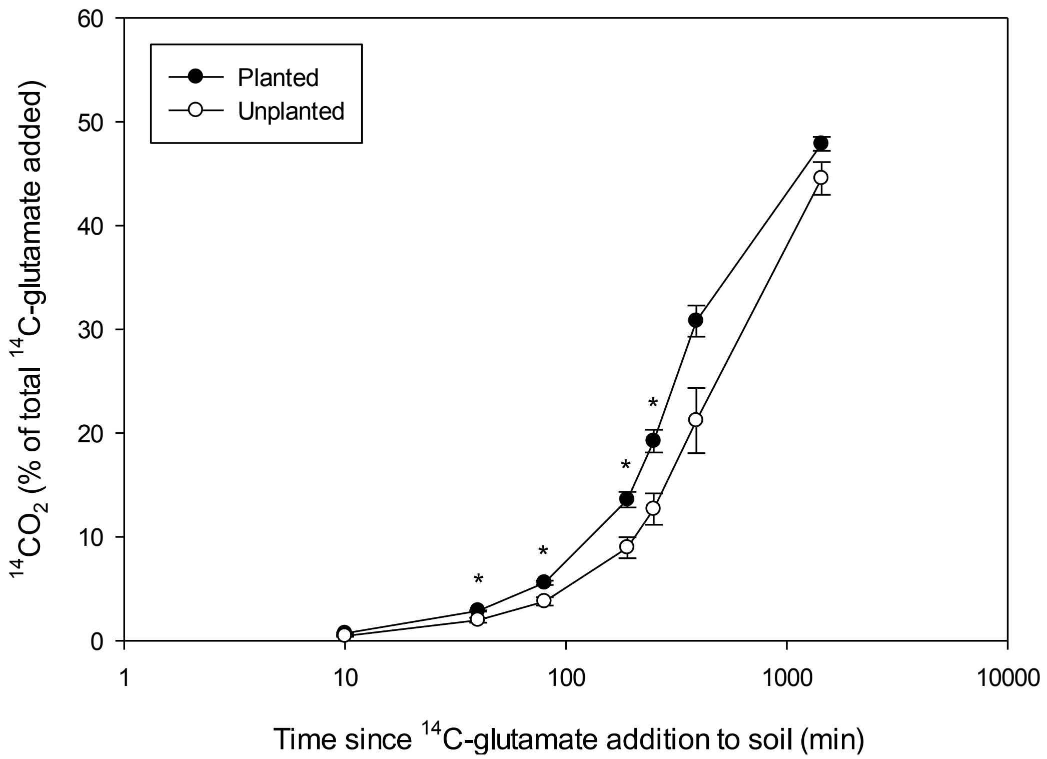

Fig. S3 Time dependent mineralization of ^{14}C-labelled glutamate in soil in the presence and absence of wheat roots. Values represent means ± SEM (n = 4). * indicate significant differences between the planted and unplanted treatments at the $P < 0.05$ level.

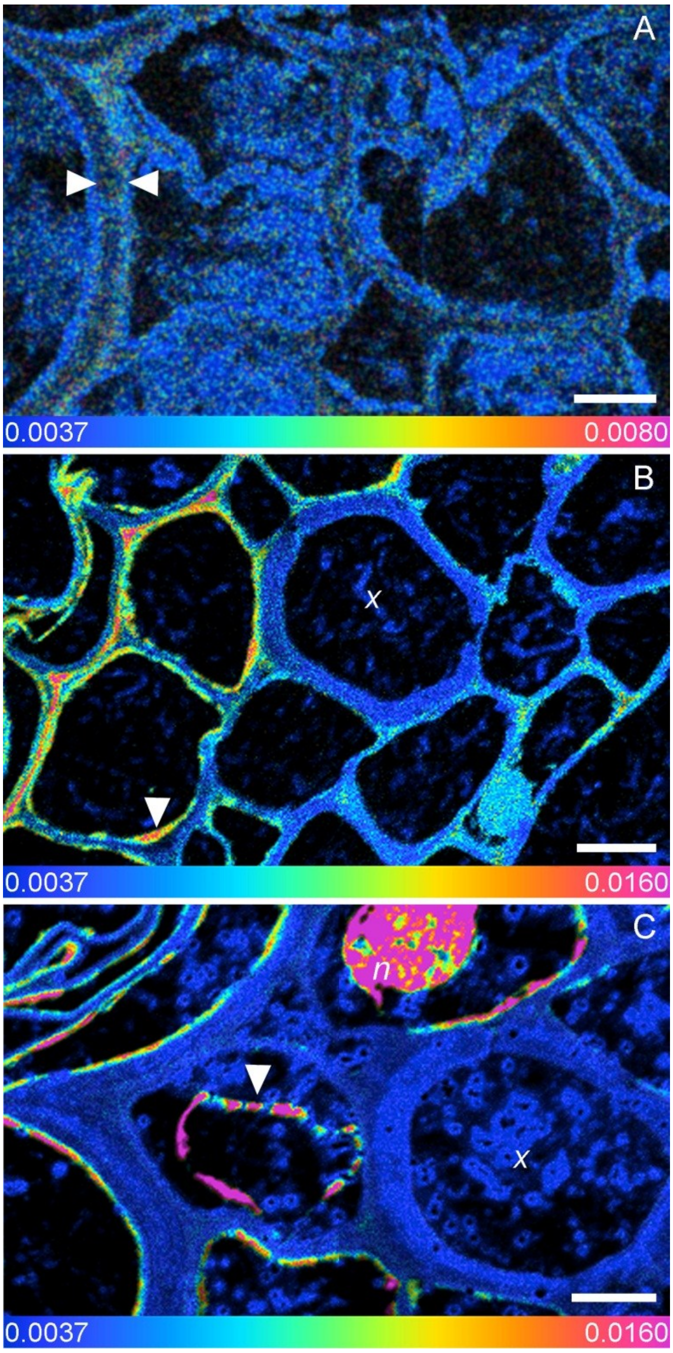

Fig. S4 NanoSIMS imaging and analysis of plant roots *in situ* within the embedded soil core after exposure to ^{15}N-labelled glutamate uptake. A) 5 min where the plant has a small amount of ^{15}N enrichment in between cell walls (between arrows), B) 30 min where the ^{15}N can be seen moving between the cell walls and in the cell cytoplasm (arrow) and C) 24 h where the ^{15}N is mainly in the cell cytoplasm (arrow) and the nucleus (n) having largely passed through the root. The xylem cells (x) remain unenriched at all times. Enrichment levels of ^{15}N are shown as $^{15}N/^{14}N$ Hue Saturation Intensity (HSI) images. The HSI color scale is optimised to show ^{15}N uptake in each image. Scale bar = 5 µm for all images.

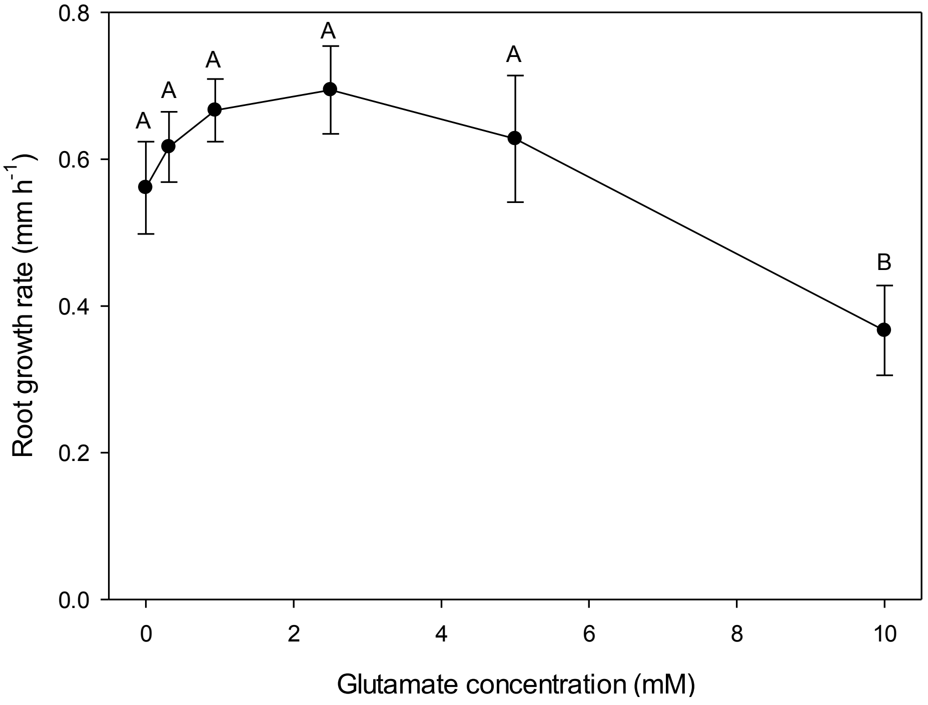

Fig. S5 Influence of external solution glutamate concentration on the growth rate of wheat roots exposed to various concentrations of glutamate for 24 h. Plants (7 d old) were grown in hydroponic culture at 20°C with a 16 h photoperiod. Alongside various concentrations of K-glutamate, the root bathing solution contained $CaCl_2$ (1 mM), KCl (1 mM) and the initial pH was adjusted to pH 6.0 with HCl. Values represent means ± SEM (n = 9). Different capital letters above the symbols represent significant differences between the concentrations at the $P < 0.05$ level. ANOVA result, $P = 0.007$. We conclude from this experiment that exogenously applied glutamate at concentrations <5 mM are not toxic to root growth over short time periods. It is therefore unlikely that the pulse addition of ^{15}N-glutamate used in our experiment (3 mM) caused any major impact on root growth.
